# Supplementary material for: Novel Formula of Antiprotozoal Mixtures
Source: Antibiotics (Basel). 2022 Jul 7;11(7):913. doi: 10.3390/antibiotics11070913 (PMC9312222; doi:10.3390/antibiotics11070913)
Supplement: Supplementary file 1 [file antibiotics-11-00913-s001.zip › antibiotics-1777636-supplementary.pdf]

# Novel formula of antiprotozoal mixtures

**Hubert Iwiński [1,3]*, Jacek Łyczko[1], Henryk Różański [2,3] and Antoni Szumny [1]**

Table of content

Figure S1. GC-MS chromatogram of tea tree essential oil ........ 1
Table S1. Composition of tea tree essential oil ........ 1
Figure S2. GC-MS chromatogram of cedar essential oil ........ 3
Table S2 Composition of Cedar essential oil ........ 3
Figure S3. GC-MS chromatogram of eucalyptus essential oil ........ 4
Table S3 Composition of eucalyptus essential oil ........ 4
Figure S4. GC-MS chromatogram of lavender oil ........ 5
Table S4. Composition of lavender essential oil ........ 5

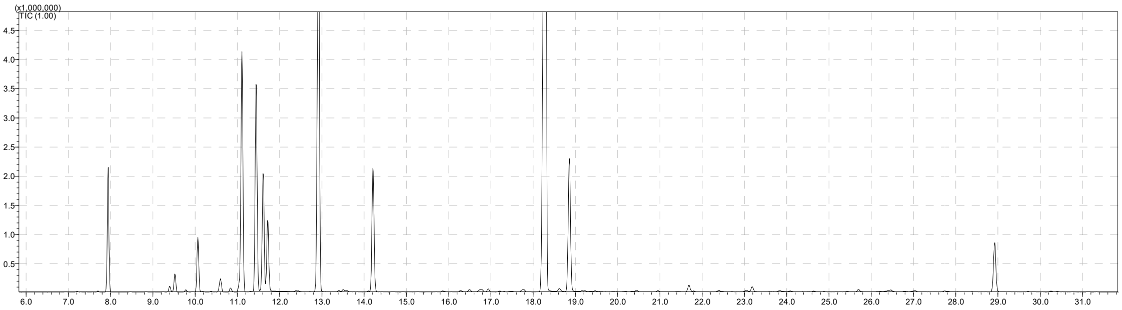


Figure S1. GC-MS chromatogram of tea tree essential oil

Table S1. Composition of tea tree essential oil

| No. | Peak Name | tR (min) | KI exp. | KI Adams | KI NIST | CAS | Area (%) |
|---|---|---|---|---|---|---|---|
| 1 | Hexadienol | 7.208 | 915 | | 916 | 17102-64-6 | 0.032 |
| 2 | α-Thujene | 7.700 | 931 | | 929 | 2867-05-2 | 0.03 |
| 3 | α-Pinene | 7.950 | 938 | 939 | 938 | 80-56-8 | 3.835 |
| 4 | Sabinene | 9.400 | 977 | | 974 | 3387-41-5 | 0.189 |
| 5 | b-Pinene | 9.533 | 980 | 979 | 979 | 127-91-3 | 0.606 |
| 6 | p-Menth-2-ene | 9.775 | 986 | | 984 | 5256-65-5 | 0.084 |
| 7 | β-Myrcene | 10.058 | 992 | | 991 | 123-35-3 | 1.77 |
| 8 | Carene <δ-2-> | 10.383 | 999 | | 1001 | 554-61-0 | 0.035 |
| 9 | α-Phellandrene | 10.600 | 1005 | | 1005 | 99-83-2 | 0.518 |
| 10 | 3-Carene | 10.842 | 1012 | | 1010 | 498-15-7 | 0.159 |
| 11 | α-Terpinene | 11.108 | 1019 | | 1017 | 99-86-5 | 8.994 |
| 12 | p-Cymene | 11.442 | 1028 | | 1024 | 99-87-6 | 7.624 |
| 13 | Limonene | 11.608 | 1032 | | 1031 | 5989-27-5 | 3.966 |
| 14 | Eucalyptol | 11.725 | 1035 | | 1032 | 470-82-6 | 2.263 |

| | | | | | | | |
|---|---|---|---|---|---|---|---|
| 15 | Ocimene | 12.392 | 1051 | | 1050 | 3779-61-1 | 0.062 |
| 16 | γ-Terpinene | 12.917 | 1063 | | 1060 | 99-85-4 | 15.384 |
| 17 | p-Mentha-3,8-diene | 13.400 | 1073 | | 1075 | 586-67-4 | 0.033 |
| 18 | cis-Linalool oxide | 13.508 | 1075 | | 1069 | 5989 - 33 - 3 | 0.053 |
| 19 | Cresol <para-> | 13.583 | 1077 | | 1073 | 106 - 44 - 5 | 0.02 |
| 20 | p-Mentha-2,4(8)-diene | 14.100 | 1088 | | 1085 | 586-63-0 | 0.013 |
| 21 | Terpinolene | 14.208 | 1090 | | 1086 | 586 - 62 - 9 | 4.827 |
| 22 | Dihydrocitronellal | 15.858 | 1128 | | 1125 | 5988 - 91 - 0 | 0.053 |
| 23 | Terpin-3-en-1-ol | 16.283 | 1138 | | 1136 | 586 - 82 - 3 | 0.067 |
| 24 | cis-b-Terpineol | 16.492 | 1142 | | 1140 | 7299-41-4 | 0.112 |
| 25 | Camphor | 16.775 | 1149 | | 1144 | 464-49-3 | 0.186 |
| 26 | Isopulegol | 16.942 | 1152 | | 1150 | 7786-67-6 | 0.121 |
| 27 | unknown | 17.208 | 1158 | | | | 0.036 |
| 28 | Isoborneol | 17.500 | 1164 | | 1165 | 10385 - 78 - 1 | 0.046 |
| 29 | Thujol | 17.775 | 1170 | | 1169 | 21653 - 20 - 3 | 0.157 |
| 30 | 1-Terpinen-4-ol | 18.250 | 1180 | | 1177 | 562-74-3 | 39.239 |
| 31 | Cymen-8-ol | 18.625 | 1188 | | 1189 | 1197 - 01 - 9 | 0.134 |
| 32 | γ-Terpineol | 18.858 | 1192 | | 1197 | 586-81-2 | 5.576 |
| 33 | trans-Dihydro carvone | 19.167 | 1198 | 1201 | 1200 | 147-86-02 | 0.054 |
| 34 | unknown | 19.467 | 1205 | | | | 0.038 |
| 35 | neoiso-Dihydro carveol | 20.450 | 1229 | | 1230 | 18675 - 34 - 8 | 0.089 |
| 36 | Isobornyl formate | 20.958 | 1240 | | 1240 | 1200 - 67 - 5 | 0.054 |
| 37 | Cinnamyl alcohol<Z-> | 21.683 | 1257 | | 1261 | 4510 - 34 - 3 | 0.253 |
| 38 | Carvone oxide<cis-> | 21.800 | 1259 | | 1262 | 18383 - 49 - 8 | 0.014 |
| 39 | Isopulegyl acetate | 22.400 | 1272 | | 1273 | 89 - 49 - 6 | 0.094 |
| 40 | neo-Isopulegyl acetate | 23.042 | 1286 | | 1278 | 109010 - 10 - 8 | 0.061 |
| 41 | Isobornyl acetate | 23.183 | 1289 | | 1287 | 125 - 12 - 2 | 0.214 |
| 42 | Terpinyl acetate <trans-dihydro-α-> | 23.833 | 1302 | | 1300 | 20777-41-7 | 0.068 |
| 43 | a-Terpinyl formate | 24.083 | 1308 | | 1306 | 2153 - 26 - 6 | 0.04 |
| 44 | Dihydrocarvyl acetate | 24.642 | 1322 | | 1325 | 20405 - 60 - 1 | 0.03 |
| 45 | a-Terpinyl acetate | 25.700 | 1347 | | 1349 | 80 - 26 - 2 | 0.13 |
| 46 | Cyclosativene | 26.467 | 1365 | | 1367 | 30541 - 92 - 5 | 0.205 |
| 47 | Longicyclene | 27.033 | 1377 | | 1372 | 1137 - 12 - 8 | 0.076 |
| 48 | 7-Epi-Sesquithujene | 27.742 | 1393 | | 1389 | 159407 - 35 - 9 | 0.081 |
| 49 | Caryophyllene | 28.933 | 1422 | | 1419 | 87-44-5 | 2.235 |
| 50 | Humulene | 30.258 | 1455 | | 1454 | 6753-98-6 | 0.039 |

tR – retention time; KI exp.– Kovats index experimental, KI Adams– Kovats index according to Adams library, KI NIST – Kovats index according to NIST library

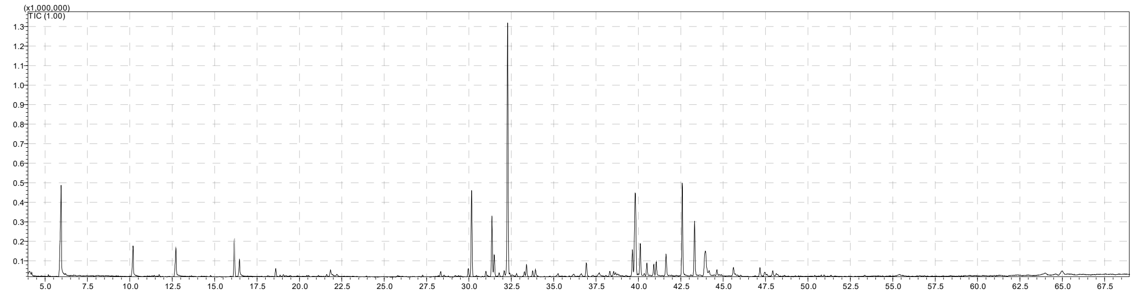

Figure S2. GC-MS chromatogram of cedar essential oil

Table S2 Composition of Cedar essential oil

| No. | Peak Name | tR (min) | KI exp. | KI Adams | KI NIST | CAS | Area (%) |
|---|---|---|---|---|---|---|---|
| 1 | Limona ketone | 16.167 | 1135 | 1131 | 1137 | 6090-09-1 | 3.656 |
| 2 | cia-b-terpineol | 16.467 | 1142 | 1144 | 1144 | 7299-41-4 | 2.072 |
| 3 | Melilotal | 18.608 | 1187 | 1188 | 1183 | 122 - 00 - 9 | 0.887 |
| 4 | *trans*-Myrtanol | 21.833 | 1260 | | 1262 | 15358-91-5 | 0.687 |
| 5 | Vestitenone | 29.967 | 1448 | | 1448 | 69401-36-1 | 0.859 |
| 6 | α-Himachalene | 30.158 | 1452 | | 1499 | 3853-83-6 | 9.072 |
| 7 | Thujopsadiene <cis-> | 31.000 | 1473 | | 1467 | 51446 - 91 - 4 | 0.456 |
| 8 | Himachalene <gamma- | 31.492 | 1484 | | 1484 | 53111 - 25 - 4 | 1.859 |
| 9 | b-Himachalene | 32.283 | 1502 | | 1500 | 1461-03-6 | 27.350 |
| 10 | Himachalene <alpha-dehydro-ar-> | 32.808 | 1517 | | 1517 | 78204 - 62 - 3 | 0.354 |
| 11 | Himachalene <gamma-dehydro-ar-> | 33.400 | 1532 | | 1530 | 51766 - 65 - 5 | 1.148 |
| 12 | g-Vetivenene | 33.758 | 1541 | | 1532 | 28908 - 26 - 1 | 0.638 |
| 13 | Bisabolene <(E)-, alpha-> | 33.925 | 1546 | | 1541 | 25532 - 79 - 0 | 0.936 |
| 14 | Himachalene epoxide | 35.258 | 1579 | | 1579 | 64825 - 84 - 9 | 0.183 |
| 15 | b-Himachalene oxide | 36.925 | 1623 | | 1619 | 57819 - 73 - 5 | 1.733 |
| 16 | Cedren-13-ol<8-> | 39.642 | 1695 | | 1689 | 18319 - 40 - 9 | 2.234 |
| 17 | Atlantone<(Z)-gamma-> | 39.808 | 1699 | | 1694 | 108549 - 48 - 0 | 12.261 |
| 18 | Atlantone<(E)-gamma-> | 40.108 | 1708 | | 1707 | 108549 - 47 - 9 | 3.222 |
| 19 | Atlantone <(Z)-alpha-> | 40.500 | 1720 | | 1716 | 56192 - 70 - 2 | 1.363 |
| 20 | Eremophilone | 40.908 | 1731 | | 1733 | 562 - 23 - 2 | 1.126 |
| 21 | Tumerone | 41.050 | 1735 | | 1734 | 180315-67-7 | 1.463 |
| 22 | Cyclocolorenone | 41.625 | 1752 | | 1757 | 489 - 45 - 2 | 2.564 |
| 23 | Atlantone <trans-, alpha-> | 42.583 | 1778 | | 1775 | 32207 - 08 - 2 | 9.638 |
| 24 | Acetoxyelemol<8-alpha-> | 43.308 | 1798 | | 1793 | 256332 - 39 - 5 | 6.188 |
| 25 | Longifolol acetate | 43.950 | 1818 | | 1820 | 205697 - 15 - 0 | 4.841 |
| 26 | Khusinol acetate | 44.433 | 1835 | | 1828 | 78405 - 34 - 2 | 0.620 |
| 27 | Flourensadiol | 45.608 | 1867 | | 1670 | 55812 - 89 - 0 | 0.796 |
| 28 | Farnesyl acetone | 47.167 | 1913 | | 1913 | 1117-52-8 | 1.135 |
| 29 | Acetoxyeudesman-4-alpha-ol <11-> | 47.925 | 1937 | | 1940 | 67996 - 33 - 2 | 0.658 |

tR – retention time; KI exp.– Kovats index experimental, KI Adams– Kovats index according to Adams library, KI NIST – Kovats index according to NIST library

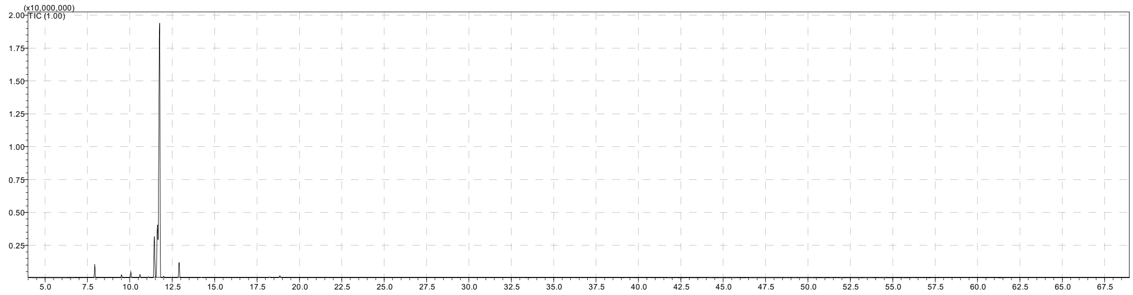


Figure S3. GC-MS chromatogram of eucalyptus essential oil

Table S3 Composition of eucalyptus essential oil

| No. | Peak Name | tR (min) | KI exp. | KI Adams | KI NIST | CAS | Area (%) |
|---|---|---|---|---|---|---|---|
| 1 | α-Pinene | 7.950 | 938 | 939 | 938 | 80-56-8 | 2.522 |
| 2 | b-Pinene | 9.533 | 980 | 979 | 979 | 127-91-3 | 0.576 |
| 3 | β-Myrcene | 10.058 | 992 | | 991 | 123-35-3 | 1.203 |
| 4 | α-Phellandrene | 10.600 | 1005 | | 1005 | 99-83-2 | 0.715 |
| 5 | .α-Terpinene | 11.100 | 1019 | | 1017 | 99-86-5 | 0.184 |
| 6 | p-Cymene | 11.450 | 1028 | | 1025 | 99-87-6 | 9.831 |
| 7 | Eucalyptol | 11.725 | 1035 | | 1032 | 470-82-6 | 80.665 |
| 8 | trans-β-Ocimene | 12.442 | 1052 | | 1049 | 3779-61-1 | 0.091 |
| 9 | γ-Terpinene | 12.908 | 1062 | | 1060 | 99-85-4 | 3.367 |
| 10 | trans-Linalool oxide (furanoid) | 14.225 | 1090 | | 1086 | 34995-77-2 | 0.142 |
| 11 | 1-Terpinen-4-ol | 18.250 | 1180 | | 1177 | 562-74-3 | 0.218 |
| 12 | γ-Terpineol | 18.858 | 1192 | | 1197 | 586-81-2 | 0.486 |

tR – retention time; KI exp.– Kovats index experimental, KI Adams– Kovats index according to Adams library, KI NIST – Kovats index according to NIST library

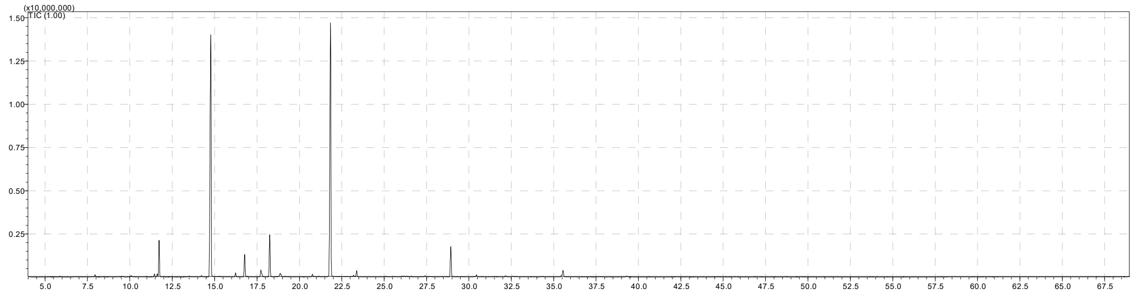

Figure S4. GC-MS chromatogram of lavender oil

Table S4. Composition of lavender essential oil

| No. | Peak Name | tR (min) | KI exp. | KI Adams | KI NIST | CAS | Area (%) |
|---|---|---|---|---|---|---|---|
| 1 | α-Pinene | 7.95 | 938 | 939 | 938 | 80-56-8 | 0.196 |
| 2 | b-Pinene | 9.533 | 980 | 979 | 979 | 127-91-3 | 0.063 |
| 3 | p-Cymene | 11.45 | 1028 | | 1025 | 99-87-6 | 0.312 |
| 4 | Limonene | 11.617 | 1032 | | 1030 | 138-86-3 | 0.249 |
| 5 | Eucalyptol | 11.725 | 1035 | | 1032 | 470-82-6 | 4.029 |
| 6 | cis-Linalool oxide | 13.525 | 1076 | | 1074 | 5989-33-3 | 0.166 |
| 7 | trans-Linalool oxide (furanoid) | 14.225 | 1090 | | 1086 | 34995-77-2 | 0.156 |
| 8 | Linalool | 14.775 | 1101 | | 1099 | 78-70-6 | 37.262 |
| 9 | 1-Octen-3-yl-acetate | 15.292 | 1114 | | 1111 | 2442-10-6 | 0.098 |
| 10 | 1,2-Dihydrolinalool | 16.233 | 1136 | 1135 | 1120 | 18479-51-1 | 0.498 |
| 11 | Camphor | 16.775 | 1149 | | 1144 | 464-49-3 | 2.955 |
| 12 | Borneol | 17.725 | 1169 | | 1166 | 464-45-9 | 1.271 |
| 13 | 1-Terpinen-4-ol | 18.25 | 1180 | | 1177 | 562-74-3 | 5.461 |
| 14 | γ-Terpineol | 18.858 | 1192 | | 1197 | 586-81-2 | 0.531 |
| 15 | trans-Dihydro carvone | 19.167 | 1198 | 1201 | 1200 | 09.04.5948 | 0.09 |
| 16 | Fenchyl acetate | 20.767 | 1236 | 1234 | 1234 | 13851-11-1 | 0.312 |
| 17 | Butanoic acid, 3-methyl-, hexyl ester | 21.158 | 1245 | 1244 | 1244 | 10032-13-0 | 0.096 |
| 18 | Linalyl acetate | 21.833 | 1260 | | 1257 | 115-95-7 | 38.875 |
| 19 | Isopulegol acetate | 23.2 | 1289 | | 1285 | 57576-09-7 | 0.206 |
| 20 | Lavandulol acetate | 23.375 | 1292 | | 1290 | 25905-14-0 | 0.728 |
| 21 | Hexyl tiglate | 25.117 | 1334 | | 1330 | 16930-96-4 | 0.101 |
| 22 | 8-Hydroxylinalool* | 25.508 | 1343 | | 1361 | 64142-78-5 | 0.089 |
| 23 | unknown | 26.05 | 1355 | | | | 0.104 |
| 24 | Neryl acetate | 26.583 | 1367 | | 1364 | 141-12-8 | 0.087 |
| 25 | Geranyl acetate | 27.417 | 1386 | | 1382 | 105-87-3 | 0.178 |
| 26 | Caryophyllene | 28.933 | 1422 | | 1419 | 87-44-5 | 4.244 |
| 27 | (E)-β-Famesene | 30.45 | 1459 | | 1457 | 18794-84-8 | 0.227 |
| 28 | Bicyclogermacrene | 32.158 | 1499 | | 1500 | 24703-35-3 | 0.164 |
| 29 | Lavandulyl isovalerate | 32.608 | 1511 | | 1508 | 51117-21-6 | 0.138 |
| 30 | γ-Cadinene | 32.808 | 1517 | | 1514 | 39029-41-9 | 0.084 |
| 31 | Germacrene B | 34.35 | 1557 | | 1559 | 15423-57-1 | 0.094 |
| 32 | Caryophyllene oxide | 35.55 | 1586 | | 1581 | 1139-30-6 | 0.729 |
| 33 | tau-Cadinol | 37.733 | 1645 | | 1642 | 147-47-90 | 0.071 |
| 34 | α-Bisabolol | 39.3 | 1686 | | 1684 | 515-69-5 | 0.136 |

tR – retention time; KI exp.– Kovats index experimental, KI Adams– Kovats index according to Adams library, KI NIST – Kovats index according to NIST librar
